# Anterolateral Thigh Chimeric Flap: An Alternative Reconstructive Option to Free Flaps for Large Soft Tissue Defects

**DOI:** 10.3390/jcm12216723

**Published:** 2023-10-24

**Authors:** Yoon Jae Lee, Junnyeon Kim, Chae Rim Lee, Jun Hyeok Kim, Deuk Young Oh, Young Joon Jun, Suk-Ho Moon

**Affiliations:** 1 Department of Plastic and Reconstructive Surgery, Yeouido St. Mary’s Hospital, College of Medicine, The Catholic University of Korea, Seoul 07345, Republic of Korea; yoonjae@catholic.ac.kr (Y.J.L.); hyeoggy@gmail.com (J.H.K.); 2 Department of Plastic and Reconstructive Surgery, Seoul St. Mary’s Hospital, College of Medicine, The Catholic University of Korea, Seoul 06591, Republic of Korea; sweety4512@naver.com (J.K.); chaerim.lee@gmail.com (C.R.L.); ohdeuk1234@hanmail.net (D.Y.O.); joony@catholic.ac.kr (Y.J.J.)

**Keywords:** perforator flap, reconstructive surgical procedures, anteromedial thigh flap, vastus lateralis, microsurgery

## Abstract

The anterolateral thigh (ALT) skin flap provides abundant, thin, pliable skin coverage with adequate pedicle length and calibre, and tolerable donor site morbidity. However, coverage of relatively large defects using the ALT flap alone is limited. We present our experience of using the ALT flap coupled with the vastus lateralis (VL) flap supplied by the same pedicle for large defect reconstruction. Between 2016 and 2020, ten patients with extensive lower-extremity or trunk defects were treated using the ALT/VL chimeric flap. The ALT portion was used to cover the cutaneous and joint defect while the VL part was used to resurface remnant defects, and a skin graft was performed. All flaps were based on the common descending pedicle, and branches to separate the components were individually dissected. All defects were successfully reconstructed using the ALT/VL chimeric flap. No surgery-related acute complications were observed, and the patients had no clinical issues with ambulation or running activities during the long-term follow-up period. With the separate components supplied by a common vascular pedicle, the ALT/VL chimeric flap allows us to reconstruct extensive defects with joint involvement or posterior trunk lesions. Thus, the ALT/VL chimeric flap may be a suitable alternative for extensive tissue defect reconstruction.

## 1. Introduction

An extensive resection of complex traumatic wounds or tumours often causes large soft tissue defects [1,2,3]. A one-stage reconstruction of large soft tissue defects is imperative to restore function and achieve aesthetic results.

Conventional reconstructive methods often rely on the use of free flaps, such as the latissimus dorsi (LD) flap and transverse rectus abdominis muscle (TRAM) flap, which have served as foundational techniques for addressing large soft tissue defects [4,5,6,7,8]. The LD flap, a reliable option, provides substantial tissue coverage but is associated with post-operative seroma formation and potential functional limitations in the arm [6]. On the other hand, the TRAM flap is limited to patients without a history of abdominal surgery or liposuction [9]. The need for a complete defect coverage may necessitate multiple free flap transfers, introducing increased complexity and associated risks [10,11].

Various muscle flaps, such as the gracilis flap, rectus abdominis flap, and gluteus maximus flap can be used in soft tissue reconstruction [12]. These flaps offer a generous volume of tissue for coverage and can be especially useful in cases where muscle function preservation is not a primary concern. Other perforator flaps, such as the deep inferior epigastric perforator (DIEP) flap and superior gluteal artery perforator (SGAP) flap, minimize muscle sacrifice while providing a reliable source of well-vascularized tissue. Local flaps, including rotational, advancement, and island flaps, are valuable options for smaller soft tissue defects [12,13]. Tissue expansion involves the gradual stretching of the existing skin to create additional tissue. It is useful for patients with limited donor sites and has been successfully employed in breast reconstruction and scar revision. In situations where an autologous tissue is not available or suitable, allografts or xenografts can be used for wound coverage. These options are often applied in cases of extensive burns or non-healing wounds [14,15,16].

The anterolateral thigh (ALT) flap has several beneficial characteristics that make it a favourable donor site for soft-tissue reconstruction of various parts of the body. These preferable characteristics include large amounts of thin, pliable skin coverage, a long vascular pedicle, convenient pedicle calibre, and minimal donor site morbidity [17,18,19,20].

However, there are some limitations in using the ALT flap alone for large defects. As advances in microsurgery have led to the development of various chimeric pattern flaps, we propose a chimeric perforator flap design in which the ALT perforator flap and vastus lateralis (VL) chimeric muscle flap are microsurgically constructed as chimeric perforator flaps to overcome the limitations of using an ALT flap alone. In addition, in the case of perforation injury during harvesting for the ALT flap, the VL muscle flap can be used as an alternative.

Although the ALT/VL muscle chimeric flap has been used to reconstruct extensive hand and neck injuries [21], studies regarding the use of this flap in the reconstruction of large trunk and lower extremity defects are limited.

In this case series, we present our experience with the ALT/VL muscle chimeric flap based on perforators from the descending branch of the lateral circumflex vessels in the reconstruction of large defects.

## 2. Patients and Methods

Ten patients who underwent an ALT/VL chimeric free flap in extensive lower extremity or trunk lesions—from January 2016 to December 2020—were analysed.

### Operative Technique

With a hand-held Doppler, mapping of the ALT perforator(s) was routinely performed before flap elevation. The operation was performed under general anaesthesia with the patients in the supine position. The ALT perforator axis—the line connecting the lateral border of the patella and anterior superior iliac spine (ASIS)—was drawn, and based on this axis, the locations of the perforator were identified using the hand-held Doppler. To design the skin island, a template of the defect was drawn, and the perforators were included in the flap design. A pinch test was used to evaluate the feasibility of primary closure at the donor site. The flap was elevated starting from the medial border. An incision was made into the deep fascia, and the intermuscular septum was identified. After a blunt dissection of the space between the rectus femoris muscle and the VL muscle using the fingers, the descending branch of the lateral circumflex artery (LCFA) was located. After confirming the origin of the perforator vessels, a careful dissection was carried out along the perforators to the skin paddle, while saving all the branches to the VL muscle flap. Various sizes of the VL muscle were harvested with the ALT flap, depending on the ideal reconstruction of various defects. The skin and muscular components of the flap—each provided by a separate branch—can be easily placed into the defect thanks to the mobility of the pedicle.

**Figure 1:**
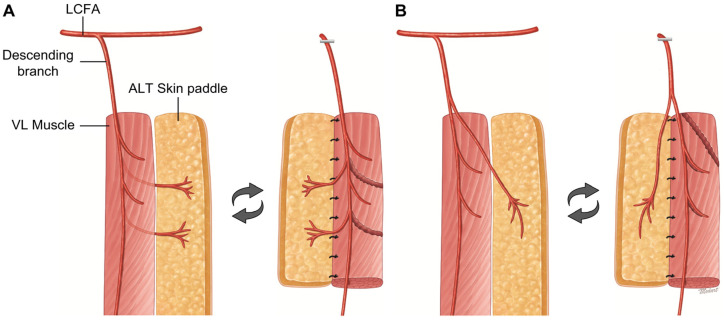
Schematic illustration of an anterolateral thigh (ALT)/vastus lateralis (VL) chimeric flap. (**A**) The musculocutaneous perforator to ALT flap is dissected at the intramuscular level between the VL muscles. The VL muscle flap is elevated on the muscular branch of the same vessel. (**B**) The ALT flap is harvested after complete dissection of the perforator, which travels briefly through the muscle, proximal to the VL muscle. Separate pedicles are directed to the VL muscle flap.

When harvesting the muscle flap, a harmonic scalpel (Ethicon Endo-Surgery, Cincinnati, OH, USA) was used to minimize bleeding and shorten the operation time. En block elevation of the ALT skin flap and VL muscle flap was performed. The descending branch of the lateral circumflex femoral vessel was dissected further in a proximal direction. The harvested chimeric flap (Figure 1A,B), skin paddle, and muscle segment were placed side by side in parallel, and the abutting margin was secured in place with Vicryl #3-0 sutures to prevent separation. Next, the conjoined chimeric flap was transferred to the defect site and the flap was temporarily fixed for stable vascular anastomosis. After vascular anastomosis, we carefully checked that there was no pulling or twisting of the main pedicle or the pedicle to each segment. The margin of the flap was checked for its viability by observing it for fresh bleeding. A split thickness skin graft was performed to cover the VL muscle segment. The flap was clinically monitored using the refilling test and clinical evaluation of the skin paddle flap colour and temperature. Between the fourth and fifth postoperative day, the split thickness skin graft was opened and checked to confirm whether the graft was successful. From week 1 after surgery, the patients were mobile.

## 3. Results

Ten patients were included in the present study; their characteristics are summarized in Table 1. Their ages ranged from 16 to 63 years, with an average of 47.7 years. Five participants were male and five were female. The aetiology was trauma (*n* = 3), tumour (*n* = 6), and infection (*n* = 1); the locations included the lower leg (*n* = 3), trunk (*n* = 5), and knee joint (*n* = 2).

The flap remained 100% viable in all patients. All defects were fully covered by the ALT/VL chimeric flap, and donor sites were closed by a primary closure in all patients. Two patients underwent radiotherapy after surgery. Hospital discharge occurred between 16 and 21 days after surgery with a mean hospitalization time of 18 days. All patients were followed up at 6 months and 1 year. The average width of the ALT flaps harvested from the ten patients was 8.6 cm (6–10 cm). The average width of the VL flaps harvested from the ten patients was 9.0 cm (7–10 cm). The average pedicle length was 7.1 cm (5–10 cm). In one case, a vein graft was necessary to elongate the pedicle length.

No major complications were encountered. One case had a minor graft site complication that resulted in unstable hypertrophic scarring on the split-thickness skin of the VL muscle area. One patient experienced flap bulkiness, which improved after subsequent liposuction. The patients did not experience any difficulty with walking or running. The results were satisfactory at the last follow-up.

### 3.1. Case Reports

#### 3.1.1. Case 1

**Figure 2:**
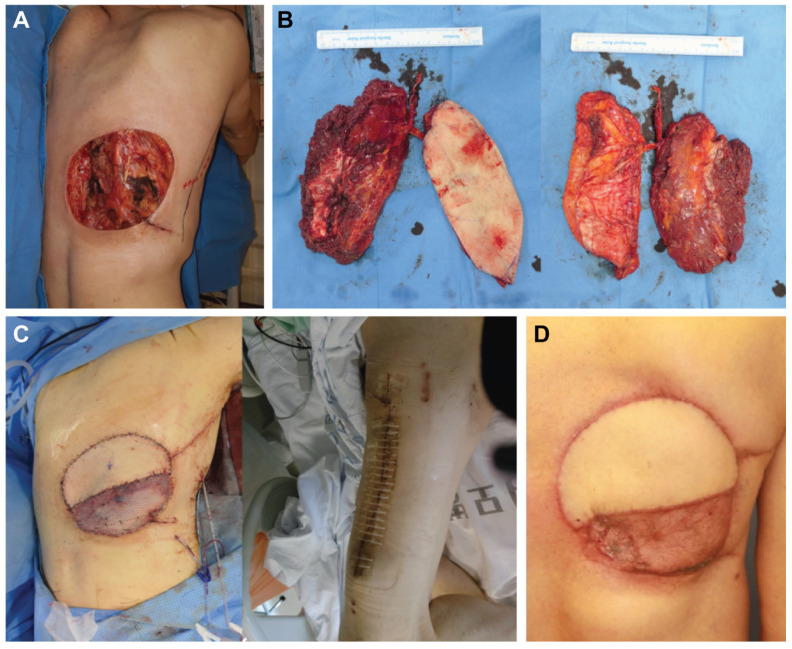
(Case 1). (**A**) Large defect size (16 × 15 cm) on the posterior trunk lesion after wide excision. (**B**) Harvested anterolateral thigh (ALT)/vastus lateralis (VL) chimeric flap. (**C**) Flap inset and immediate postoperative photo of the donor site. (**D**) Clinical photo at 4 months post-operation.

A 63-year-old male patient presented with a recurrent known malignant peripheral nerve sheath tumour infiltrating the posterior trunk region. Under general anaesthesia and in the prone position, the lesion was resected with a 3 cm margin. The size of the resultant defect was 16.0 × 15.0 cm (Figure 2A) with rib exposure. For defect reconstruction, the patient was placed in the supine position and the ALT/VL chimeric flap was elevated with skin and muscular components of 6.0 × 16.0 and 10.0 × 15.0 cm, respectively (Figure 2B). The autologous vein graft was harvested to elongate the pedicle length from the great saphenous vein. Defects were reconstructed as described previously, and the patient healed uneventfully (Figure 2C,D).

#### 3.1.2. Case 2

**Figure 3:**
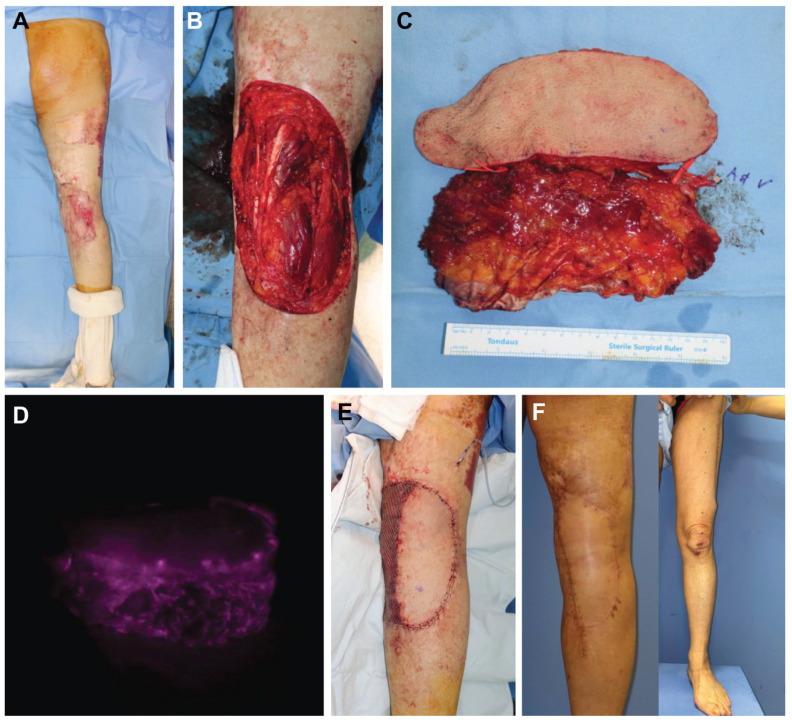
(Case 2). (**A**) Preoperative clinical photo before wide excision. (**B**) Extensive defect size (15 × 20 cm) on the right popliteal area. (**C**) Harvested anterolateral thigh (ALT)/vastus lateralis (VL) chimeric flap. (**D**) The Indocyanine Green Angiography (ICG) fluoroscopy of the harvested flap shows stable illumination of both the ALT skin flap and VL muscle flap. (**E**) Postoperative clinical photo immediately after ALT/VL flap coverage. (**F**) Clinical photo at 12 months post-operation. The flap was well incorporated, and the donor site healed well.

A 63-year-old female presented with a Marjolin’s ulcer originating from the right popliteal area (Figure 3A). A wide excision was performed, leaving an extensive defect size of 15.0 × 20.0 cm (Figure 3B). An ALT/VL chimeric flap was harvested from the left thigh with skin and muscular components of 7.0 × 20.0 and 8.0 × 18.0 cm, respectively (Figure 3C). The skin paddles and muscular components for the flaps were placed side by side to cover the skin surface of the soft-tissue defect of the right knee. The donor sites were closed directly. After anastomosis, an intraoperative Indocyanine Green Angiography (ICG) study showed that the flap circulation was intact (Figure 3D). All flaps survived completely. The recipient site presented a satisfactory contour (Figure 3E,F). The patient was able to ambulate fully with no apparent functional deficits related to the donor site at their last follow-up visit 12 months after the operation.

#### 3.1.3. Case 3

**Figure 4:**
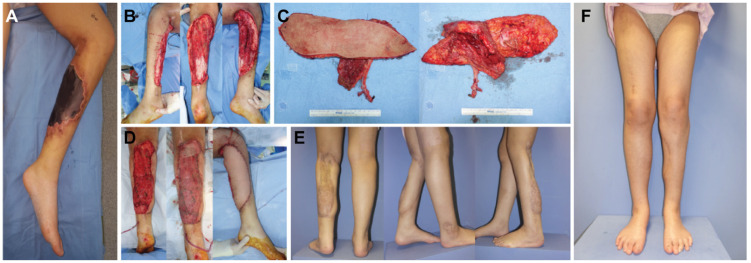
(Case 3). (**A**) Skin and soft tissue necrosis involving the right lower leg. (**B**) Large defect size (37 × 19 cm) after radical debridement. (**C**) Harvested anterolateral (ALT)/vastus lateralis (VL) chimeric flap. (**D**) Immediate postoperative clinical photo. (**E**,**F**) Postoperative clinical photo at 12 months post-operation.

A 17-year-old female patient suffered from leukaemia that caused skin and soft tissue necrosis in the right lower leg (Figure 4A). After radical debridement, the resultant defect was 37.0 × 19.0 cm with tibial exposure (Figure 4B). An ALT/VL chimeric flap was microsurgically harvested to reconstruct the extensive defect in one stage. The skin paddle of the flap and the muscle component dimensions were 37.0 × 8.0 cm and 20.0 × 8.0 cm, respectively (Figure 4C). The skin paddle of the flaps and muscle components were placed side by side to cover the defect. Above the muscle component, a split-thickness skin graft was performed (Figure 4D). The postoperative course was uneventful. The recipient site showed a satisfactory contour and mild bulkiness of the flap site led to considerations for possible fat injection for contour correction in the future (Figure 4E).

## 4. Discussion

The present study analysed a series of ten cases of reconstruction for complex large defects using the ALT/VL chimeric flap in one stage. We demonstrated that this is a safe and reliable alternative option for the reconstruction of large defects in various cases.

Traditionally, various free flaps, such as the LD, TRAM, and ALT, have been used for the reconstruction of extensive large defects [9,22,23,24,25,26]. Among these flaps, the ALT free flap is well known and has been used as a standard flap due to its advantages, such as the easy anatomical approach to pedicles and relatively easy harvesting [6,7,23]. However, when the defect is very large, the ALT flap alone may not be able to cover the defect.

In general, the ALT/VL chimeric flap cannot be selected as the first choice for wide defect coverage. However, it may be selected as an alternative option in the following cases. First, this method may be used when TRAM or LD flaps are contraindicated or when patients refuse a donor-site scar in the abdomen or trunk area. Second, this method may be used in cases where an ALT flap alone is planned initially, but the defect area is larger than expected and the ALT flap alone is inadequate to cover the defect. Harvesting the wider skin flap is possible in the ALT, but in this case, an additional microanastomosis process of turbocharging or supercharging must be performed to incorporate anteromedial thigh (AMT) perforators into the flap. Third, this method may be used in cases where the defect area is less sensitive than the donor site of thigh lesions, such as trunk lesions.

A chimeric perforator flap consisting of independent tissue flaps, such as skin flaps and muscle flaps with their own independent vascular supply linked to a common vascular source, has many advantages in covering extensive tissue defects [6,27,28,29,30,31,32].

The ALT flap is a perforator and intermittent septocutaneous flap provided by the lateral cutaneous perforator of the descending branch of the LCFA, which is a branch of the deep femoral artery [1,4,17]. The VL muscle is a type I muscle predominantly supplied by the same descending branch of the ALT flap, although it can also be fed by the transverse branches of the LCFA [21,22,33]. This vascular anatomy enables the ALT and VL muscle flaps to be elevated as a chimeric flap.

The advantages of harvesting the ALT/VL chimeric flaps to reconstruct extensive tissue defects are as follows. First, the chimeric flap provides a large amount of soft tissue and multiple flap components that an individual flap cannot provide; therefore, extensive defects can be covered using the chimeric flap in one stage [6,31,33]. Second, donor site primary closure is possible, which yields better aesthetic results and minimizes donor site morbidity. Third, the ALT/VL chimeric flap can be elevated simultaneously without the need for patient repositioning. Moreover, only one pair of recipient vessels is required to supply the entire chimeric flap. When we compare the ALT/VL chimeric flap with the ALT turbocharged flap with an AMT perforator [23], no additional microsurgical anastomosis is necessary; therefore, this technique consumes less time, and a more straightforward flap harvesting is possible.

Although the ALT/VL chimeric flap has many benefits in the reconstruction of extensive tissue defects, there are several disadvantages to this method. First, the use of the ALT/VL chimeric flap requires a longer learning curve and technical difficulty is high. Second, this technique requires the coverage of a skin graft on top of the VL muscle flap, which may cause contour deformity and create an aesthetically unfavourable outcome. Third, the ALT flap presents variable anatomy [19,20,23], where muscular dissection is necessary in most cases, increasing the operation time. In addition, there may be no perforator vessels arising from the descending branch of the lateral circumflex to the skin flap. However, even in these cases, it is possible to harvest a chimeric flap based not on perforators but on the entire descending branch of the LCFA with the segment of the VL muscle as needed.

In this study, we focused on assessing the outcomes of the chimeric flap technique for the reconstruction of large soft tissue defects. However, it is important to note that we did not directly compare these outcomes with those of conventional large skin flaps of the ALT under identical conditions. This represents a significant limitation of our study, as a direct comparative analysis would have provided valuable insights into the relative advantages and disadvantages of these two surgical approaches. Further studies with a large patient group and long-term follow-up are needed to assess the effectiveness of this technique.

## 5. Conclusions

The novel ALT/VL chimeric flap is a safe, effective, and well-tolerated method with acceptable donor site morbidity. This makes the ALT/VL chimeric flap a useful alternative for the reconstruction of wide extensive defects in various cases. In our study, no major complications were observed, with encouraging functional and aesthetic outcomes.

## Figures and Tables

**Table 1:** Demographic information of patients who underwent ALT & VL chimeric flap.

Patient No.	Gender	Age	Diagnosis	Location	ALT Size (cm)	VL Size (cm)	Total Flap Size (cm)	Pedicle Length (cm)
1	M	63	Sarcoma	Trunk	16 × 6	15 × 10	27 × 20	5
2	F	63	SCC	Knee joint	14 × 8	18 × 10	26 × 25	5
3	F	16	Infection	Lower leg	14 × 8	10 × 8	22 × 18	7
4	M	58	SCC	Lower leg	15 × 10	11 × 8	25 × 18	9
5	F	38	Trauma	Trunk	13 × 9	10 × 9	23 × 18	6
6	M	28	Scar contracture	Knee joint	15 ×10	11 × 10	20 × 15	8
7	F	44	Trauma	Lower leg	15 ×10	10 × 9	25 × 10	6
8	M	32	DFSP	trunk	11 × 7	9 × 7	18 × 14	8
9	F	63	Fibrosarcoma	Trunk	13 × 10	10 × 9	23 × 17	10
10	M	72	Osteosarcoma	Trunk	12 × 8	13 × 10	25 × 16	7
Average		47.7						7.1

ALT, anterolateral thigh; VL, vastus lateralis; SCC, squamous cell carcinoma; DFSP, dermatofibromasarcoma protuberans.

## Data Availability

The data can be obtained by scientists who conduct work independently from the industry, on request. The data are not stored on publicly available servers.
